# Synthetic B-Cell Epitopes Eliciting Cross-Neutralizing Antibodies: Strategies for Future Dengue Vaccine

**DOI:** 10.1371/journal.pone.0155900

**Published:** 2016-05-25

**Authors:** Babu Ramanathan, Chit Laa Poh, Kristin Kirk, William John Hannan McBride, John Aaskov, Lara Grollo

**Affiliations:** 1 Research Centre for Biomedical Sciences, Sunway University, Kuala Lumpur, Malaysia; 2 Swinburne University of Technology, Melbourne, Australia; 3 James Cook University Clinical School, Cairns Base Hospital, Cairns, Queensland, Australia; 4 Queensland University of Technology, Brisbane, Queensland, Australia; 5 Australian Catholic University, Melbourne, Australia; University of Malaya, MALAYSIA

## Abstract

Dengue virus (DENV) is a major public health threat worldwide. A key element in protection from dengue fever is the neutralising antibody response. Anti-dengue IgG purified from DENV-2 infected human sera showed reactivity against several peptides when evaluated by ELISA and epitope extraction techniques. A multi-step computational approach predicted six antigenic regions within the E protein of DENV-2 that concur with the 6 epitopes identified by the combined ELISA and epitope extraction approach. The selected peptides representing B-cell epitopes were attached to a known dengue T-helper epitope and evaluated for their vaccine potency. Immunization of mice revealed two novel synthetic vaccine constructs that elicited good humoral immune responses and produced cross-reactive neutralising antibodies against DENV-1, 2 and 3. The findings indicate new directions for epitope mapping and contribute towards the future development of multi-epitope based synthetic peptide vaccine.

## Introduction

Dengue virus (DENV) is a major public health problem especially in the tropical and subtropical regions of the world with approximately 390 million people infected annually [1]. DENV comprises four serotypes (DENV-1, 2, 3, and 4) which belong to the *Flavivirus* genus of the *Flaviviridae* family. The DENV genome is composed of a single, positive-stranded RNA genome of 11 kb that codes for a large polyprotein comprising a capsid protein (C), a membrane protein (M), the major envelope glycoprotein (E) and other non-structural proteins [2].

The E protein is involved in receptor binding of DENV and is the target of neutralising antibodies. The E protein ectodomain consists of three structural domains referred to as domain I (EDI), domain II (EDII), and domain III (EDIII) [3]. EDI is the central domain containing virus-specific cross-reactive epitopes [4]. EDII contains the fusion loop and is involved in dimerization and membrane fusion. The highly conserved fusion loop forms the epicentre of a series of overlapping immunodominant cross-reactive epitopes eliciting predominantly non- or weakly neutralizing antibodies [5, 6]. EDIII is an immunoglobulin-like structure that contains DENV complex cross-reactive epitopes with neutralizing antibodies to multiple serotypes [7, 8].

Dengue infections can vary from asymptomatic or self-limiting mild flu-like illness to classical dengue fever (DF), to the more severe disease state characterized as dengue hemorrhagic fever (DHF) and dengue shock syndrome (DSS) [9]. The severe complications are reported to be due to the pathogenic manifestations of the complex human immune responses, antibody cross-reactivity leading to disease enhancement due to cytokines and chemokines [10, 11]. A number of vaccine candidates are under development such as live attenuated vaccines, chimeric vaccines, recombinant vaccines, inactivated vaccines, virus like particles and subunit vaccines [1, 12]. The Sanofi Pasteur tetravalent chimeric yellow-fever dengue (CYD-TDV) vaccine (Dengvaxia^®^) is the front-runner of all experimental vaccines after completing a double-blinded, placebo-control, large phase III clinical trial in Asia (Indonesia, Malaysia, Philippines, Thailand, Vietnam) [13] and the Latin America (Brazil, Colombia, Honduras, Mexico, Puerto Rico) [14]. CYD-TDV was created by inserting the DENV pre-M and E genes in to the cDNA backbone of the YF 17D vaccine, replacing the native yellow fever pre-M and E genes. Although the overall vaccine efficacies in Asia and Latin America were reported to be 56.5% and 64.7%, respectively, the serotype-specific vaccine efficacy in Asia was substantially lower at 50% for serotype 1 and 35% for serotype 2 [13]. Similar trend in serotype-specific vaccine efficacy was also reported in the Latin American phase III clinical trial where the efficacies were 50.3% for serotype 1 and 42.3% for serotype 2 [14].

Epitope identification through the use of short synthetic peptides has drawn much attention and a number of synthetic peptide-based approaches have identified the antigenic determinants in DENV [15–18]. Computational biology has contributed to predictive pathobiology of life threatening organisms and there are many bioinformatics tools that can be applied to predict the B and T cell epitopes [19]. A number of attempts were made to predict the B-cell epitopes of DENV with improvements in the accuracy of B-cell epitope prediction by designing more appropriate algorithms such as the Hidden Markov Model (HMM) [20] and the Artificial Neural Network (ANN) [17, 21–23].

Proteolytic footprinting methods such as the epitope extraction technique has been used to map the epitopes of human immunodeficiency virus (HIV) and hepatitis C virus (HCV) [24, 25]. This approach is unique in that it is able to identify the antigen bound to the antibody in its native conformation under physiological conditions [24, 25]. The detection of the antigen is possible in combination with matrix-assisted laser desorption (MALDI)-time of flight (TOF) mass spectrometry for the characterization of linear and discontinuous epitopes. However, B-cell epitope prediction using a single method is usually not sufficient to identify epitopes at a scale greater than random and a multiple step epitope identification scheme can help to increase the odds of identifying novel candidate epitopes. In addition, short synthetic peptides generally do not induce good immune responses on their own and helper T-cell can be provided through co-synthesizing linear helper T-cell epitopes along with the B-cell epitopes [26].

In this study, we combined different strategies for B-cell epitope identification and evaluate the vaccine potency of synthetic peptide based vaccine constructs in mice. A multi-step sequence and structure based bioinformatics approach was used to predict the potential B-cell epitope candidates. Also, an overlapping peptide library representing the entire E protein of DENV-2 was used to screen the anti-dengue Immunoglobulin G (IgG) purified from neutralising DENV-2 polyclonal human sera. The binding profile of each peptide against the IgG was evaluated using both ELISA and epitope extraction approach. A combination of ELISA and epitope extraction revealed several novel linear B-cell epitopes which provide new insights for future development of epitope-based dengue vaccines. Selected peptides were co-synthesised with a known T-helper epitope from dengue virus [27] and these vaccine constructs were evaluated in mice for their potential to elicit anti-peptide antibodies that could cross-neutralise the dengue virus.

## Materials and Methods

### Ethical considerations

The study was approved by the Cairns and Hinterland Health Service District Human Research Ethics Committee (Protocol No. HREC/10/QCH/17-646), and the Human Research Ethics Committee of Swinburne University of Technology (SUHREC- 2010/158). Detailed explanation of the study was provided and written consent to participate in the study was obtained from volunteers. All data were handled confidentially. The protocol for animal use was reviewed and approved by the Swinburne Animal Ethics Committee (SAEC) (Protocol No. SAEC Project 2011/02).

### Human sera

The subjects/travelers were recruited based on their previous infection history and hospital records held at Cairns Base Hospital, Queensland, Australia. The presence of dengue antibody was identified using PanBio dengue IgM/IgG kit (Brisbane, Australia) and the infected serotype was determined by RT-PCR during the acute infection stage. For this study, we collected the convalescent sera from dengue positive volunteers and stored at -20°C until further use. Focus reduction neutralisation test (FRNT) was performed to determine the neutralising ability of the patient sera. The sera that showed positive neutralisation against one or more DENV serotypes were included in the study. The negative control samples did not neutralise any DENV serotype and was negative to dengue IgG ELISA. The subjects exposed to other flaviviruses or flavivirus vaccines were excluded based on hospital records. The FRNT titers of sera against all 4 dengue serotypes and other particulars of the subjects are provided as supplementary (S1 Table).

### Cell lines, viruses and antibodies

Baby hamster kidney cells (BHK-21 clone 15) [28], *Aedes albopictus* mosquito cells (C6/36) [29], dengue virus prototypes DENV-1 (Hawaii), DENV-2 New Guinea C (NGC), DENV-3 (H87) and DENV-4 (H241) were kind gifts from Professor John Aaskov, WHO collaborating Centre for Arbovirus Reference and Research, Queensland University of Technology, Australia.

Stocks of DENV prototype viral strains were grown using confluent monolayer of C6/36 cells. Plaque titration was performed on BHK-21 cells as previously described by Morens et al. (1985) with modifications. Briefly, cell monolayers were prepared by seeding 24 well tissue culture plates and infected with serial ten-fold dilutions (10^−1^ to 10^−6^) of virus in RPMI-1640 (Invitrogen, MA, USA) containing 2% heat-inactivated FBS (Invitrogen, MA, USA). After 2 hours, the wells were overlaid with 1.5% w/v CMC overlay medium and incubated at 37°C with 5% CO_2_ for 5–6 days. The infected cells positive for the E protein of DENV were enumerated by intracellular staining using the monoclonal antibody 4G2 (clone D1-4G2-4-15) (Chemicon, Merck Millipore, MA, USA) which was recognized by Horseradish Peroxidase (HRP)-conjugated goat anti-mouse IgG (Sigma, MO, USA).

### Immunoglobulin G purification

The immunoglobulin G (IgG) fractions were purified from human sera using Protein A-Sepharose Fast Flow affinity chromatography (Sigma-Aldrich, MO, USA) following manufacturer’s instructions.

### Log Neutralization Index (LNI)

The neutralizing ability of purified IgG from human sera was measured by a constant antibody-varying virus plaque-reduction neutralization test [28]. Briefly, BHK-21 cell monolayers were prepared in 24 well tissue culture plates with 1 ml of cells at a concentration of 2 x 10^5^ cells/ml in RPMI-1640 (Invitrogen, MA, USA) containing 2% heat-inactivated FBS. DENV serotypes 1–4 with a working stock of approximately 10^6^ PFU/ml were diluted ten-fold (10^−1^ to 10^−6^) in RPMI-1640 containing 2% heat-inactivated FBS. A concentration of 10 μg/ml of purified human IgG was diluted in RPMI-1640 containing 2% heat-inactivated FBS and 100 μl was mixed with an equal volume of serially diluted viral suspensions in a 96 well round bottom plate. For a negative control, 100 μl of RPMI 1640 was mixed with equal volume of viral suspension.

Following incubation at 37°C with 5% CO_2_ for 2 hours, the virus-antibody mixture was added in duplicate to the monolayers of confluent BHK-21 cells and incubated at 37°C with 5% CO_2_ for 1 hour to allow the virus to absorb to the cells. The wells were then overlaid with 1 ml of 1.5% CMC (Sigma-Aldrich, MO, USA) and RPMI-1640 containing 2% heat-inactivated FBS. The plates were incubated at 37°C with 5% CO_2_ for 4–5 days. After incubation, the cells were fixed with 3.7% formaldehyde (Sigma-Aldrich, MO, USA), permeabilised with 0.1% Nonidet P40 (Sigma-Aldrich, MO, USA) and blocked with 2% w/v skimmed milk (Nestle, WA, Australia). The monoclonal antibody 4G2 was used to label the infected cells and was visualized using horseradish peroxidase (HRP)-conjugated rabbit anti-mouse IgG (Sigma-Aldrich, MO, USA). The viral foci were counted and the virus titre was recorded as FFU/ml. Virus neutralization was indicated by the reduction in virus titre following the addition of purified IgG to virus culture. Neutralization was expressed as a Log Neutralization Index (LNI).

### Focus reduction neutralization test (FRNT)

The neutralizing antibody titres of immune mice sera were determined by FRNT as previously described [30]. Pooled pre-immunization or post-immunization mice sera were heat-inactivated at 56°C for 30 min and diluted 1:10 with RPMI-1640 containing 2% heat-inactivated FBS. Serial two-fold dilutions of this inactivated mice sera were prepared and 100 μl of the dilutions were mixed with an equal volume of virus suspension containing 50 FFU (focus forming units) per well. Following incubation at 37°C for 2 hours, the virus-antibody mixture was added in duplicate to the monolayers of confluent BHK-21 cells and incubated at 37°C with 5% CO_2_ for 2 hour to allow the virus to absorb to the cells. The wells were then overlaid with 1.5% w/v CMC (Sigma-Aldrich, MO, USA) and incubated for 4–5 days. After incubation, the cells were fixed with 3.7% formaldehyde (Sigma-Aldrich, MO, USA), permeabilised with 0.1% Nonidet P40 (Sigma-Aldrich, MO, USA) and blocked with 2% w/v skimmed milk (Nestle, WA, Australia). The monoclonal antibody 4G2 was used to label the infected cells and was visualized using horseradish peroxidase (HRP)-conjugated rabbit anti-mouse IgG (Sigma-Aldrich, MO, USA). The neutralizing antibody titer was calculated as the reciprocal of the highest serum dilution that produced a 50% reduction of 50 focus forming units compared to control containing the virus and pre-immunization sera [31].

### Enzyme Linked Immunosorbent Assay (ELISA)

Antibody titres were determined by ELISA as described previously [32]. Briefly, ELISA plates were coated overnight at room temperature with 10 μg/ml of peptides or 5 μg/ml of DENV recombinant E proteins (Hawaii biotech, USA). Wells were blocked with 50 μl of purified IgG diluted to 20 μg/ml. For individual or pooled mice sera, serial half-log dilutions were prepared and 50 μl were added to each well. The plates were incubated for 1 hour and the sera or purified IgG dilutions were removed and the wells were washed twice. An aliquot of 50 μl (1:1000 dilution) of HRP-conjugated goat anti-human IgG (Sigma-Aldrich, MO, USA) or HRP-conjugated rabbit anti-mouse IgG (Sigma-Aldrich, MO, USA) was added. The optical density (OD) of individual wells was read by an iMark Microplate Reader (BioRad Laboratories, Hercules, CA, USA) at a wavelength of 450nm. Antibody titres were expressed as the reciprocal of the logarithm of that dilution of serum that gave an OD four times above that obtained in wells with pre-immune control sera.

### Epitope extraction

The extraction of bound peptides to purified human IgG was carried out as described previously by Grollo et al. (2006). A total of 70 peptides spanning the sequence of the E protein were arranged into 14 groups containing 5 peptides each at a concentration of 0.2 mg/ml in PBS. None of the 5 peptides in a group had the same molecular mass and care was taken to make sure that there was minimal or no peptide sequence overlaps in each group. To 50 μl of each peptide pool, 50 μl of purified IgG (50 μg) from either dengue patients or non-infected controls was added and allowed to react for 30 min at room temperature. The peptide-antibody mixture was then added to compact reaction columns (CRC’s) (USB, Cleveland, OH, USA) containing 50 μg of Protein A-sepharose beads in 50 μl of 50mM Tris-HCl (pH 7.4) and incubated for 30 min at room temperature. The unbound peptides were washed once with washing buffer containing 50mM Tris-HCl (pH7.4) in 0.5M NaCl and 0.5% N-octyl-D-glucoside (Calbiochem, San Diego, USA) and the peptides bound to the antibody were eluted from Protein-A sepharose beads by addition of 50 μl of elution buffer (0.5% formic acid, Fluka, Buchs, Switzerland).

### Mass spectrometry

The eluted peptides were analysed in a Bruker microFLEX (Bruker Daltonics, Germany) matrix-assisted laser desorption time-of-flight mass spectrometer (MALDI-TOF). Briefly, 1 μl of eluted peptide was dried on a MALDI sample stage (Bruker Daltonics, Germany) with 1 μl of MALDI matrix containing α-cyano-4-hydroxycinnamic acid (Sigma-Aldrich, MO, USA) in 50% acetonitrile (Sigma-Aldrich, MO, USA) and 0.1% Tri-fluro acetic acid (Sigma-Aldrich, MO, USA). Detection was performed in positive reflector mode with m/z range of 1500–3500 using the flexControl software (version 3.0, Bruker Daltonics. Germany). Peak lists were generated by the flexAnalysis software (version 3.0, Bruker Daltonics, Germany) using the Snap peak detection algorithm with default settings. Peptides were also identified using LC-ESI-MS/MS. An aliquot of 10 μl of the eluted peptides were loaded in glass vials (Waters, MA, USA) and separated by Agilent 1100 series nanoLC (Agilent Technologies, Palo Alto, CA, USA) on a 5 μm (150 mm x 75 μm) Zorbax 300SB-C18 (Agilent Technologies, Palo Alto, CA, USA) chip column using ChipCube interfaced at the front end of a LC/MSD Trap XCTplus 3D iontrap mass spectrometer (Agilent Technologies, Palo Alto, CA, USA). Solvent in the mobile phase A consisted of 0.1% formic acid (Fluka, Buchs, Switzerland) in water and mobile phase B consisted of 95% acetonitrile (Sigma-Aldrich, MO, USA) with 0.1% formic acid (Fluka, Buchs, Switzerland). The elution gradient for the chip column was from 15% to 50% buffer B over 19 minutes. The mass spectrometer was operated with electrospray ionisation in the positive ion mode with m/z range of 1500–3500. Data was acquired using the 6300 Series ion Trap LC/MS Software 6.1 (Agilent Technologies, Palo Alto, CA, USA). Selected ions were subject to further MS-MS analysis to confirm peptide identity.

Alternatively, 15 μl of the eluted peptides were loaded onto a 300 μm x 5 mm Zorbax 300SB-C18 (Agilent Technologies, Palo Alto, CA, USA) reversed-phase precolumn attached to a Shimadzu Prominence nano LC system (Shimadzu Corporation, Kyoto, Japan). The precolumn was washed with 0.1% formic acid in 5% acetonitrile for 15 min before placing in-line with a 150 mm x 75 μm Zorbax 300SB-C18 (Agilent Technologies, Palo Alto, CA, USA) reversed-phase column. Peptides were eluted using a gradient of 5–65% (v/v) acetonitrile in 0.1% formic acid over 60 min, at a flow rate of 0.25 μl/min. Peptides were analyzed via electrospray ionization on a QSTAR Elite hybrid quadrupole time-of-flight (QqTOF) mass spectrometer (Applied Biosystems/MDS Sciex, Foster City, CA, USA). The mass spectrometer was operated in the positive ion mode with ion source voltage of 2,200 V. Analyst QS 2.0 software (Applied Biosystems/MDS Sciex, Foster City, CA, USA) was used to collect data in a data-dependent acquisition mode for the three most intense ions fulfilling the following criteria: m/z between 450 and 2,000; ion intensity 40 counts; and charge state between **+**2 and **+**3. After MS/MS analysis, these ions were dynamically excluded for 18 s, using a mass tolerance of 50 mDa. MS scans were accumulated for 0.5 s, and MS/MS scans were collected in automatic accumulation mode for a maximum of 2 s.

### Database search

The peptide mass fingerprints (PMFs) from MALDI-ToF were searched against a local copy of the non-redundant database SWISS-PROT (http://www.expasy.ch/sprot) using the MASCOT search program [33]. The MS/MS peak lists in mgf file format (.mgf) from the ion trap were generated using default parameters in DataAnalysis version 3.4 (Agilent Technologies, Palo Alto, CA). Peak lists for QSTAR Elite data were made using ProteinPilot^TM^ software version 3.0 (Applied Biosystems/MDS Sciex) and searched against the dengue virus database (Swissprot/Uniprot) using the Paragon algorithm [34]. The Paragon algorithm search parameters were: Sample type: identification; Cys alkylation: none; Digestion: none; Instrument: LTQ; Search effort: Thorough ID; Detected protein thresh hold: >0.05 (10%). The false positive rate determined was 0.2%.

### *in silico* epitope prediction

The epitope prediction workflow was carried out according to Kirk *et al*. (2012). A combined *in silico* approach utilizing computational hidden Markov model (HMM), propensity scale algorithm, and artificial learning was used to identify 15-mer structurally conserved B-cell epitopes on the E protein of DENV-2. The epitopes were identified by combining both sequence (ABCPred, BepiPred) and structure based (epitopia) prediction approaches. The dengue virus 2 sE protein was computationally analysed for hydrophobicity, surface accessibility, solvent accessibility, polarity and spatial distance orientation relationships. The sequences were obtained from NCBI Genbank and scored for the key antigenic characteristics against the BLASTP [35] query algorithm. Multiple publicly available database sets were sorted and aligned via the ClustalW. Conserved sequences demonstrating homology within the protein data bank listings PDB ID: lOAN and 1k4r were used to construct and verify the model. The predicted epitopes were displayed on a three dimensional structure of DENV-2 E protein (PDB ID-1OAN) using PDB viewer software. Three dimensional structural model was generated based on the structural conservation (more than 40%) with the PDB model using the Chimera [36] interface to modeler [37]. The rate of amino acid substitutions in the alignment of homologous proteins was estimated with the help of epitopia server having the reconstructed crystallographic atomic co-ordinates.

### Synthesis of peptide vaccine constructs

Seven vaccine constructs were designed consisting of B cell epitopes selected from our screening strategies. The B cell epitopes consisted of linear 18-mer synthetic peptides denoted as B2, B16, B29, B38, B45, B64 and B19. Individual linear peptides were co-synthesized with a helper T-cell epitope representing the amino acids 352- LITVNPIVTEKDSPVNIE-368 of the DENV-2 E protein (Jamaica strain). All the vaccine constructs were synthesized by attaching the T-helper epitope to the amino- terminal end of the B-cell epitope. This T_H_–cell epitope has previously been reported to enhance the antibody response to flavivirus B-cell epitopes in BALB/c mice [38].

### Mice immunization

Male 6–8 week old BALB/c mice (5 animals per group) were obtained from the Animal Facility, Department of Microbiology and Immunology, the University of Melbourne. The animal facility personnel monitored the animals daily, checking for levels of food and water in each cage. Mice were also physically checked twice weekly by the researcher. The peptide immunogen (50μg) was suspended in PBS and emulsified in a 1:1 ratio of complete Freund’s adjuvant (Sigma-Aldrich, MO, USA) for the first priming dose or incomplete Freund’s adjuvant (Sigma-Aldrich, MO, USA) for the booster dose. The animals were injected subcutaneously at the base of the tail with the vaccines in 100uL of solution. The mice were inoculated with vaccine construct on day 0 (primary dose) and the booster dose was administered on day 28. The Animal house personnel oversee the basic animal maintenance including housing the animals in cages, a palatable diet, water and bedding. A time-controlled lighting system was used to ensure a regular diurnal light cycle. The cages were cleaned and sanitised on regular basis. A protocol was in place for early/humane endpoint if animals became severely ill prior to experimental endpoint. Humane endpoints were defined as follows: If a mouse showed signs of ill health such as weight loss (greater than 20% of body weight), hunching, ruffled fur or lethargy, it will be euthanized. The researcher were to be notified immediately if the facility personnel detected mice that appear unwell. This was recorded in the diary provided. The researcher was then asked to inspect the animal and euthanize if necessary. However, none of the animals died prior to experimental endpoint and did not meet the criteria for euthanasia. We also had a protocol in place to alleviate animal suffering if needed by anesthesia. Following anesthesia, mice were monitored until they regained consciousness, at which point they were returned to their cages. The animals were bled on day 0, 10 and 38. Following the final bleed on day 38, the animals were euthanized by CO_2_ asphyxiation for 2–3 minutes in a CO_2_ chamber. Sera was centrifuged and the plasma was stored at -20°C until further use.

### Statistical analysis

Statistical analysis of all data were performed with GraphPad Prism 5 software (GraphPad Software, San Diego, CA, USA). Data derived from at least two independent assays are presented as mean ± standard error of the mean (SEM). A P value of **<**0.05 was considered statistically significant for all parameters and the confidence interval (CI) was 95%. Mean LNI’s were compared to detect significant differences between antibody titres to viruses using one-way analysis of variance (ANOVA) followed by the Bonferroni multiple comparison test. The statistical analyses of the mice antibody titres were carried out using two-tailed Student’s t–test and each of the resultant P values for a particular comparison is shown in the appropriate text or in the figure legend. Mean FRNT_50_ values were compared by one-way ANOVA followed by Tukey’s honestly significant difference (HSD) multiple comparison test with significance level alpha (P) set at 0.05.

## Results

### Identification of peptide-antibody binding through ELISA

Sera collected from dengue positive human subjects were screened against 18-mer synthetic peptides representing the DENV-2 E protein through direct binding ELISA (S2 Table). Of the total 70 peptides being screened, 17 peptides showed high reactivity towards the DENV-2 human polyclonal IgG. The IgG from four DENV negative individuals was used as a control and the mean absorbance of negative control plus 3 times the standard deviation (OD-0.128) was used as the cut-off line to identify the positive IgG reactive peptides.

**Fig 1:**
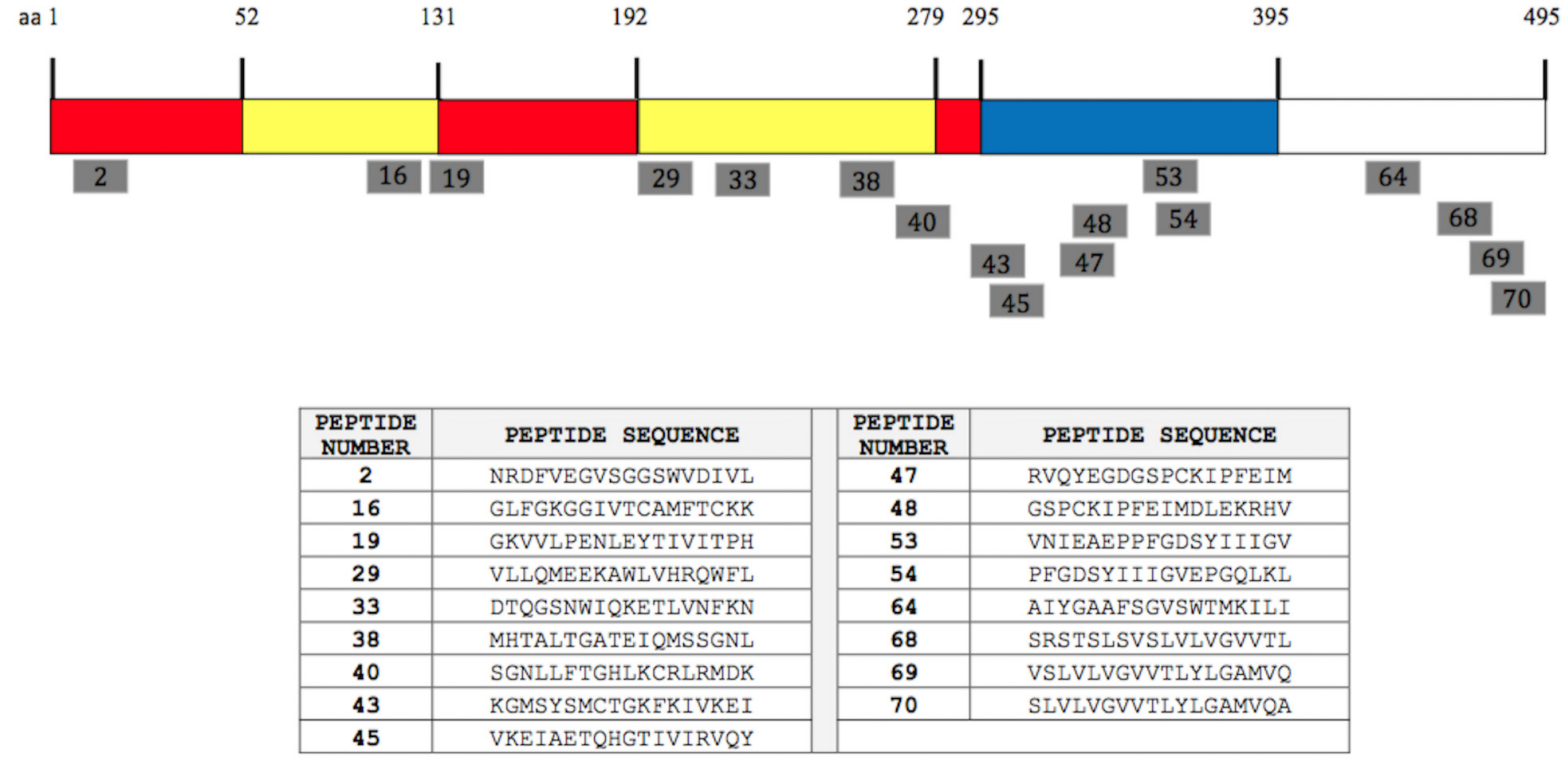
Synthetic peptides of the DENV-2 E-glycoprotein binding with IgG in ELISA. The position of the 17 peptides (18-mers) recognized by the immune sera in ELISA is shown as grey bars. The soluble E protein (residues 1–395) comprising domains I, II, and III are coloured in red, yellow, and blue, respectively. The peptide sequences for each of the synthetic peptides are shown.

The relative position of the 17 peptides representing the DENV-2 E protein is shown in Fig 1. The peptides were distributed in several regions across the entire E protein of DENV-2 at amino acid positions 8–25, 106–123, 127–144, 190–207, 225–242, 260–291, 295–347, 365–389, 442–459 and 470–495. Peptides 2 and 16 were found within the EDI and EDII of sE, respectively. Peptide 19, corresponding to aa 127–144, is located within the “hinge” region between EDI and EDII, and previous studies have shown that the partial sequence (aa 127–134) of peptide 19 is an immunodominant epitope [39]. The P40 peptide (aa 274–291) was located within the “hinge” region between EDI and EDIII, and the epitope interacting with the fusion loop reactive monoclonal antibody MAb 4G2 has been reported to be harbored in this region. MAb 4G2 is highly conformational and broadly cross-reactive to all four DENV serotypes [40].

The region corresponding to amino acids 295–347 (represented by peptides 43, 45, 47 and 48) and aa 365–389 (represented by peptide 53 and 54) are located within the EDIII domain of the sE protein. Apart from epitopes present on the soluble E protein, four peptides were identified in two different antigenic regions representing the membrane proximal “stem” region of the E ectodomain corresponding to aa 442–459 (represented by peptide 64) and aa 470–495 (represented by peptides 68, 69 and 70).

### Epitope extraction

The synthetic peptides were grouped into 14 pools with each pool comprising 5 peptides. These peptides were allowed to react with purified IgG in solution at neutral pH. The subsequent mass spectrometry of the reactive peptides showed 21 peptides able to bind to the DENV-2 positive IgG (S3 Table), whereas IgG from DENV negative human sera did not show reactivity towards these peptides. Since we used an overlapping peptide library with 11 amino acid overlaps at both ends, we selected the positively reacted peptides based on the reaction pattern. For example, since the peptides 1, 2 and 3 all reacted positive, we selected peptide 2 for animal studies as it represents 11 amino acids of peptide 1 and 3. This selection criterion was carried out in order to advance the peptides to animal studies.

**Fig 2:**
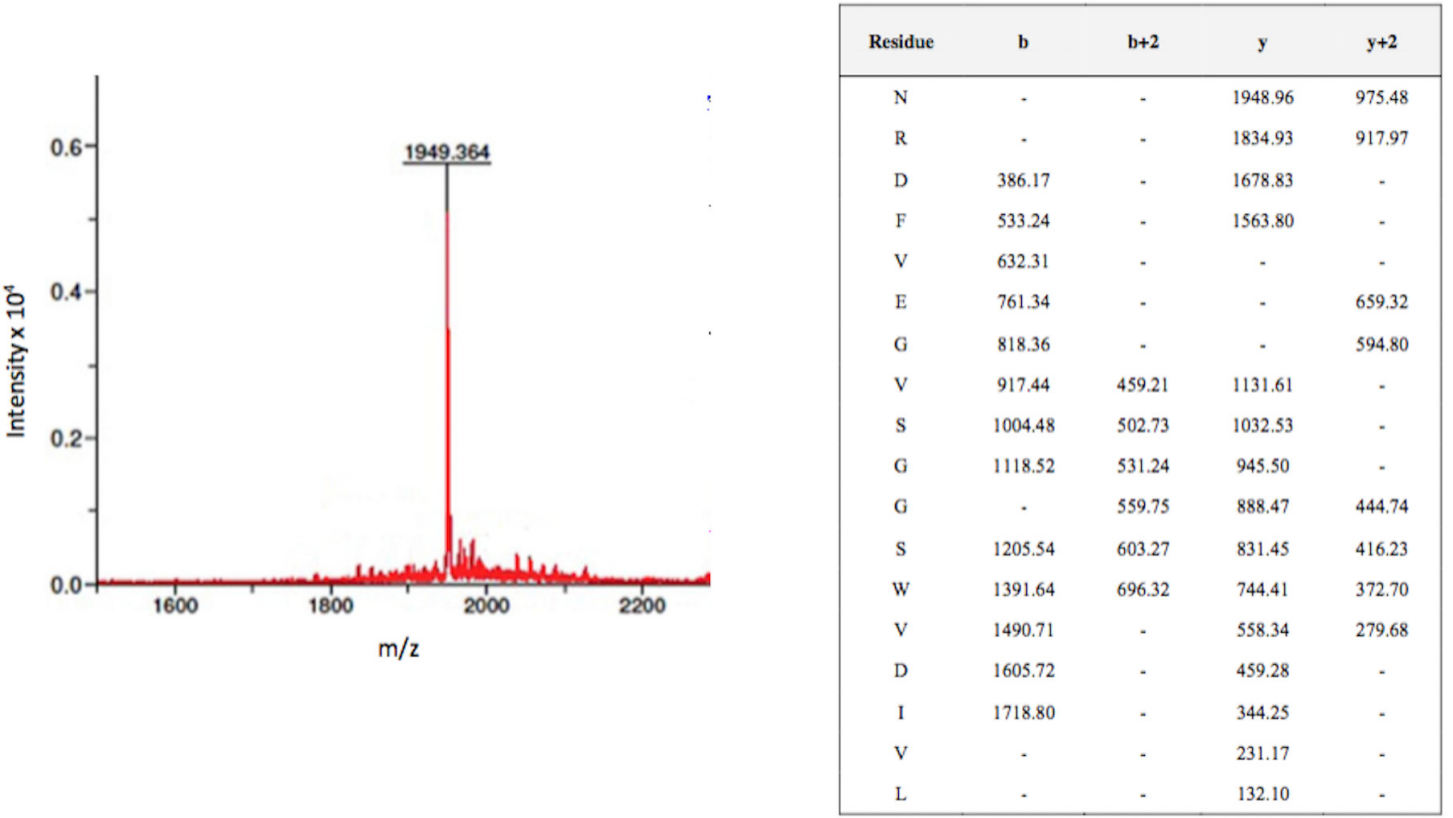
Mass spectrum of peptide 2 identified through MALDI-ToF mass spectrometry corresponding to m/z 1949. Pools of overlapping peptides (18-mers, n = 5) spanning the entire E protein of DENV-2 were assembled such that no pool contained peptides of similar mass. These peptide pools were allowed to react with IgG purified from DENV-2 infected patient’s sera and the antibody-bound peptides were extracted in acidic conditions. Following extraction, one peptide with a m/z value of 1949 was identified through MALDI-ToF mass spectrometry. MS/MS sequencing of peptide ion mass 1949 unequivocally defines it as deriving from peptide 2 sequence (NRDFVEGVSGGSWVDIVL) with the b and y ion coverage shown in the table.

For example, peptide 2 (NRDFVEGVSGGSWVDIVL) corresponding to aa 8–25 of the E protein (EDI) reacted with most immune IgGs tested. Mass spectrometry showed the corresponding m/z value of 1949 with possible b and y ion coverage both as single and/or doubly charged ion (Fig 2). We have shown the chromatogram of peptide 2 as an example representation of mass-spectrometry studies. A similar pattern of antibody reaction against all IgGs was observed with peptide 16 (106-GLFGKGGIVTCAMFTCKK-123), with a m/z value of 1861 and peptide 45 (309-VKEIAETQHGTIVIRVQY-326), with a m/z value of 2084. The immunodominant peptide 19 located in the “hinge” region between EDI and EDII (127-GKVVLPENLEYTIVITPH-144) and peptide 64 representing the membrane proximal “stem” region (442-AIYGAAFSGVSWTMKILI-459) of the E protein also showed a positive reaction against antibodies in solution with a m/z value of 2022 and 1928, respectively. In contrast to ELISA which showed non-binding to IgG, peptides 29 (197-VLLQMEEKAWLVHRQWFL-214) and 38 (260-MHTALTGATEIQMSSGNL-277) showed an ability to bind to 14 IgGs present in clinical samples when tested using the epitope extraction approach.

### *in silico* B-cell epitope prediction

**Fig 3:**
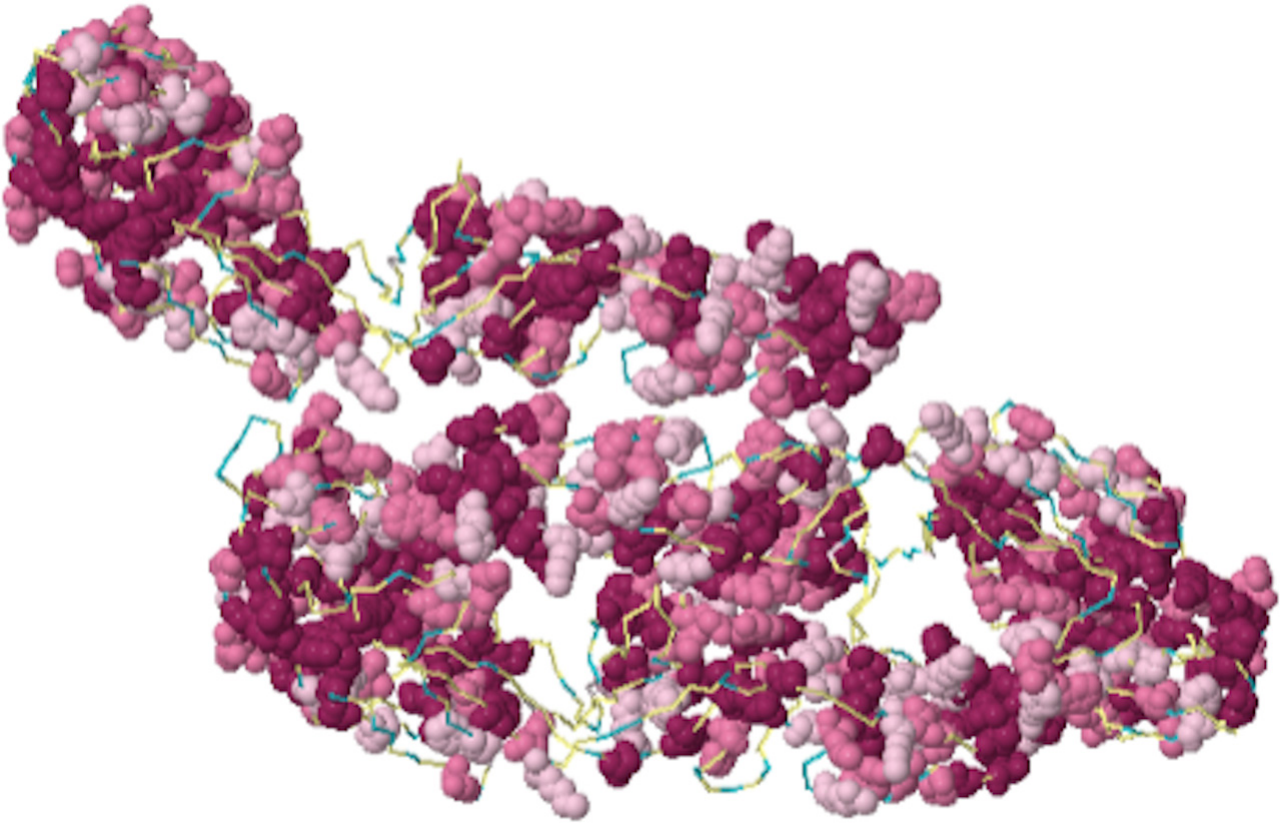
Immunogenicity of DENV E protein (PDB id: 1k4r) super imposed with Bayesian Predictive Algorithm. Top view of the homology model of DENV E protein monomer, PDB id- 1k4r is shown with amino acid residues indicated in space filling model. Colour coded regions correspond to epitopes predicted to have neutralizing ability. Areas shaded in red are indicative of most conserved, surface exposed residues and most likely immunogenic. Areas shaded in light pink are exposed and likely immunogenic, where as those in blue are considered least conserved and not immunogenic.

A multi-step computational approach was used to identify the linear B-cell epitopes from the DENV-2 E protein based on surface accessibility of residues, hydrophobicity and the spatial distance orientation relationship [26]. The potential linear epitopes identified through the computational approach were shown as 14-mer amino acid sequences. A score of > 0.65 was considered as the cut off level for selecting positive epitopes. There were 16 B-cell epitopes identified on the E protein of DENV-2 (Table 1). The epitopes identified on DENV-2 E protein were categorized into 6 distinct antigenic regions. Epitopes found in greater spatial proximities were discounted as their normal position lay buried and potentially limiting exposure of the epitope on the surface. All predicted sequences of the B-cell epitopes were screened to determine if they shared any sequence similarity with human proteins and none was found to be homologous with the human proteome, thus confirming that there would be no occurrence of auto-immune reactions. Areas that are shaded in red indicate the most conserved, and most likely immunogenic based on the physiochemical properties of amino acids and their corresponding stereochemistry. Exposed and likely immunogenic regions are clustered around the underside of the canyon of this predicted model and shaded in light pink. Regions that are not considered immunogenic are shown in blue (Fig 3).

**Table 1:** Epitopes identified on the DENV-2 E protein.

Regions	Epitope sequence	Position on E protein	Score
1	RCIGISNRDFVEGV	2–1	0.89
	GISNRDFVEGVSGG	55–18	0.85
2	DRGWGNGCGLFGKG	98–111	0.80
	GLFGKGGIVTCAMF	106–119	0.75
	KGGIVTCAMFTCKK	110–123	0.69
3	EGKIVQPENLEYTI	126–139	0.82
	PENLEYTIVITPHS	132–145	0.81
4	FNEMVLLQMENKAW	193–206	0.84
	LQMENKAWLVHRQW	199–213	0.70
	LDLPLPWLPGADTQ	214–227	0.70
5	GSQEGAMHTALTGA	254–267	0.68
	TALTGATEIQMSSG	262–275	0.73
6	SYSMCTGKFKVVKE	298–311	0.81
	KVVKEIAETQHGTI	307–320	0.78
	AETQHGTIVVRVQY	313–326	0.77
	IVVRVQYEGDGSPC	320–333	0.80

The potential linear epitopes identified through the computational approach were shown as 14-mer amino acid sequences. A score of > 0.65 was considered as the cut off level for selecting positive epitopes.

### Immunogenicity of predicted B-cell epitopes

**Fig 4:**
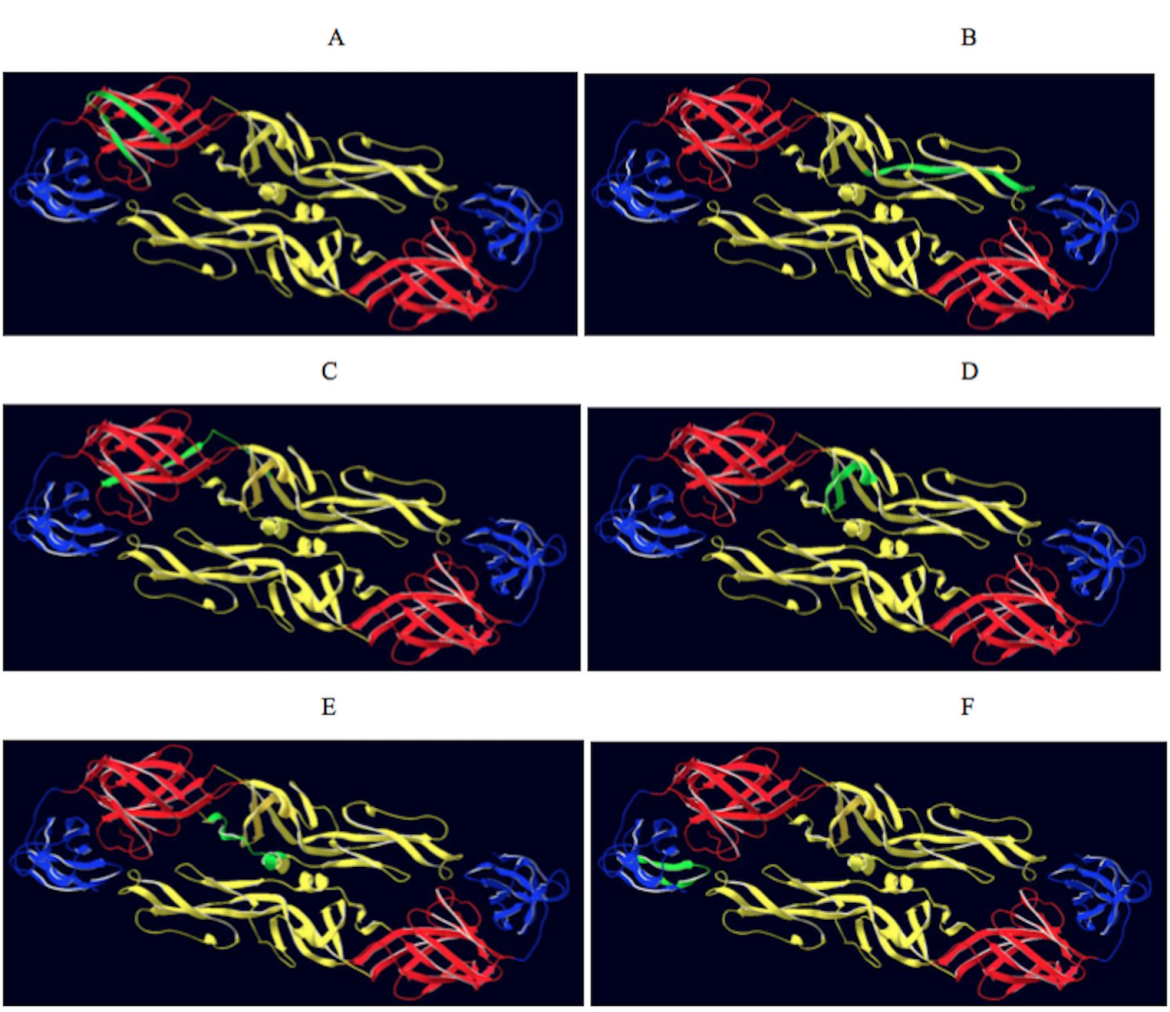
Location of epitopes on the DENV-2 protein. Top view of the dimeric form of the DENV-2 E protein residues 1–395 (PDB ID-LOAN). The domains I, II and III are coloured red, yellow and blue, respectively. The epitopes identified from 3 different epitope mapping strategies were coloured in green. A) e pitope B2, B) epitope B16, C) epitope B19, D) epitope B29, E) epitope B38, F) epitope B45.

**Fig 5:**
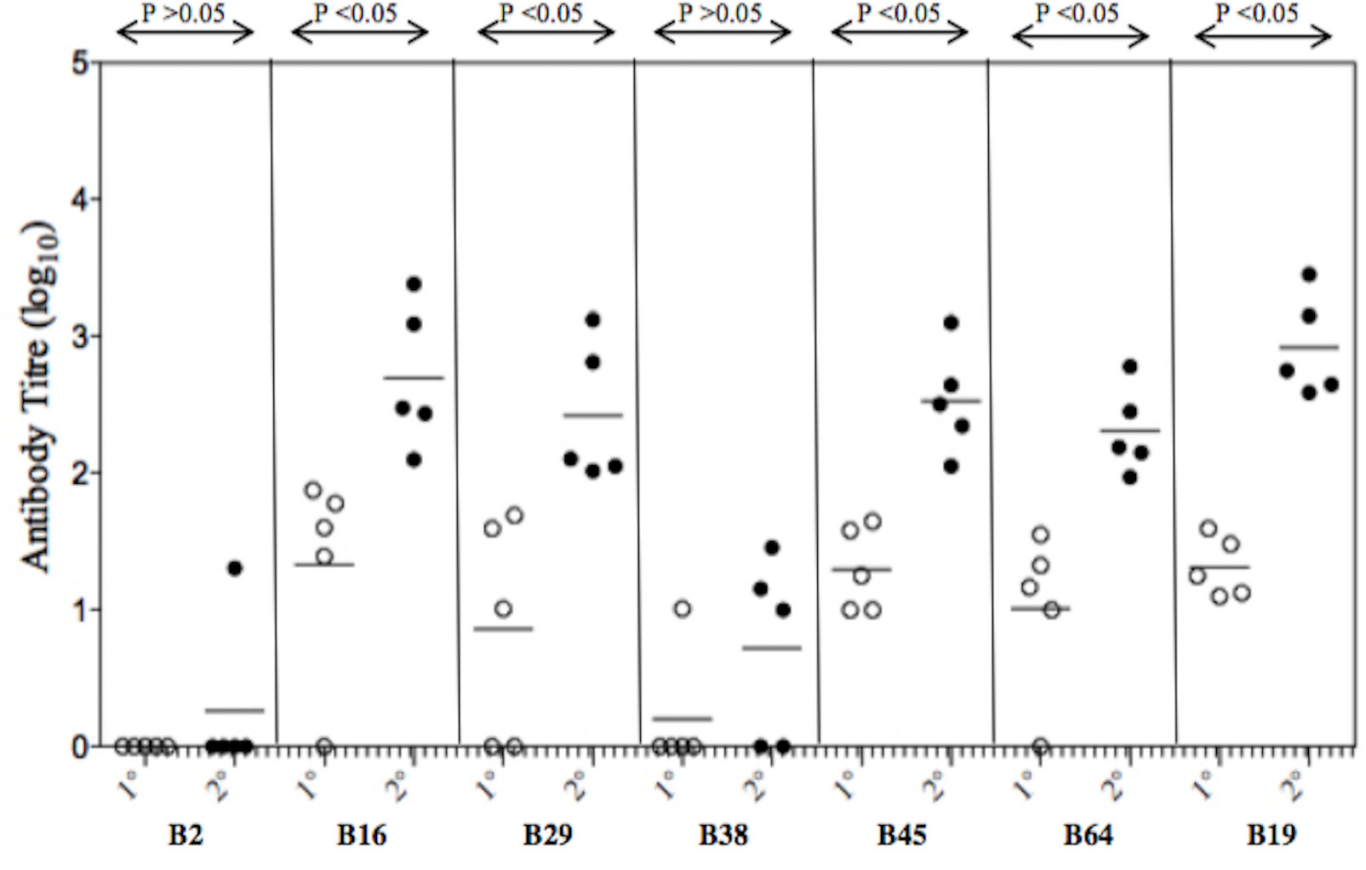
Immunogenicity of peptide vaccines B2, B16, B29, B38, B45, B64 and B19 coupled to a helper T-cell epitope. For primary (open circles) inoculation, groups of BALB/c mice (n = 5) were administered subcutaneously with 50 μg of the peptide immunogen in complete Freund’s adjuvant (CFA) on day 0 (1°). Negative control animal groups received CFA and saline. The secondary (closed circles) booster dose was administered subcutaneously on day 28 (2°) with 50 μg of the peptide immunogen in incomplete Freund’s adjuvant (IFA). Mice were bled on days 0, 10 and 38, and sera obtained. ELISA was performed using 18-mer synthetic peptide representing each of the 7 peptides as the antigen coated overnight on 96 well plates. Antibody titres are expressed as the reciprocal of the logarithm of that dilution of serum that gave an optical density four times above that obtained in wells with pre-immune control sera. Individual animal titres are presented with the mean value represented by the horizontal bar and p values are indicated between the primary and secondary dose.

A total of 7 B-cell epitopes (B2, B16, B29, B38, B45, B64 and B19) were evaluated as vaccine candidates as these epitopes were identified through a combination of three epitope mapping strategies (Table 2). The location of 6 of these epitopes is displayed on the 3-dimensional structure of the DENV-2 E protein (PDB ID- LOAN) (Fig 4). The T-helper epitopes were attached to the amino- terminal of the B-cell epitope. The resulting vaccine construct was used to inoculate the mice on day 0 (primary dose), and the booster dose was administered on day 28. The animals were bled on days 0, 10 and 38. The immune sera were used to determine the anti-peptide antibody response in ELISA using the corresponding 18-mer native B-cell epitope as the antigen. The antibody response of individual mouse within a group and the mean antibody titre of each vaccine construct are shown in Fig 5. Following the secondary booster dose, the peptides B16, B29, B45, B64 and B19 elicited significant anti-peptide antibody titres (P <0.05) when compared to the primary dose. Though the antibody titres elicited by these five peptides following secondary booster were not significantly different, the highest mean titre was observed in mice sera elicited by the vaccine construct B19 (log_10_ 2.918) followed by B16 (log_10_ 2.697). There was no statistically significant reduction observed in binding efficacy between specific antibodies to the conjugate peptide (T-helper and B-cell epitope) when compared to the native 18-mer B- cell peptide (S1 Fig).

**Table 2:** Comparison of amino acid sequences of peptides B2, B16, B29, B38, B45, B64 and B19 between the four DENV serotypes.

**Serotype**	**NCBI accession number**	**Peptides**						
		**B2**	**B16**	**B29**	**B38**	**B45**	**B64**	**B19**
		NRDFVEGVSGGSWVDIVL	GLFGKGGIVTCAMFTCKK	VLLQMEEKAWLVHRQWFL	MHTALTGATEIQMSSGNL	VKEIAETQHGTIVIRVQY	AIYGAAFSGVSWTMKILI	GKVVLPENLEYTIVITPH
DENV-1	AAZ43213.1	NRDFVEG***L***SG***AT***WVD***V***VL	GLFGKG***SLI***TCA***K***F***K***C***VT***	VLL***T***M***K***EK***S***WLVH***K***QWFL	MHTALTGATEIQ***T***S***GTTT***	***E***KE***V***AETQHGT***VLVQ***V***K***Y	***TA***YG***VL***FSGVSWTMKI***G***I	GK***I***V***QY***ENL***K***Y***SVIV***T***V***H
DENV-2	AAA17500.1	NRDFVEGVSGGSWVDIVL	GLFGKGGIVTCAMFTCKK	VLLQMEEKAWLVHRQWFL	MHTALTGATEIQMSSGNL	VKEIAETQHGTIVVRVQY	AIYGAAFSGVSWTMKILI	GKVVLPENLEYTIVITPH
DENV-3	ABY82134.1	NRDFVEG***L***SG***AT***WVD***V***VL	GLFGKG***SL***VTCA***K***F***Q***C***LE***	***I***LL***T***M***KN***KAW***M***VHRQWF***F***	MHTALTGATEIQ***T***S***G***G***TS***	***K***KE***VS***ETQHGTI***L***I***K***V***E***Y	***SA***Y***T***A***L***FSGVSW***I***MKI***G***I	GK***A***V***QH***ENL***K***YT***VI***IT***V***H
DENV-4	AEX97810.1	NRDFVEGVSGG***A***WVD***L***VL	GLFGKGG***V***VTCA***K***F***L***C***SG***	***I***L***MK***M***KK***K***T***WLVH***K***QWFL	MH***S***AL***A***GATE***VDSGD***GN***H***	***D***KE***M***AETQHGT***T***V***VK***V***K***Y	***SV***Y***TTM***F***G***GVSW***MIR***ILI	G***NL***V***QI***ENLEYT***V***V***V***T***V***H

Sequence alignments of multiple viral strains. Sequence variations observed between four DENV serotypes are indicated in bold italics.

**Fig 6:**
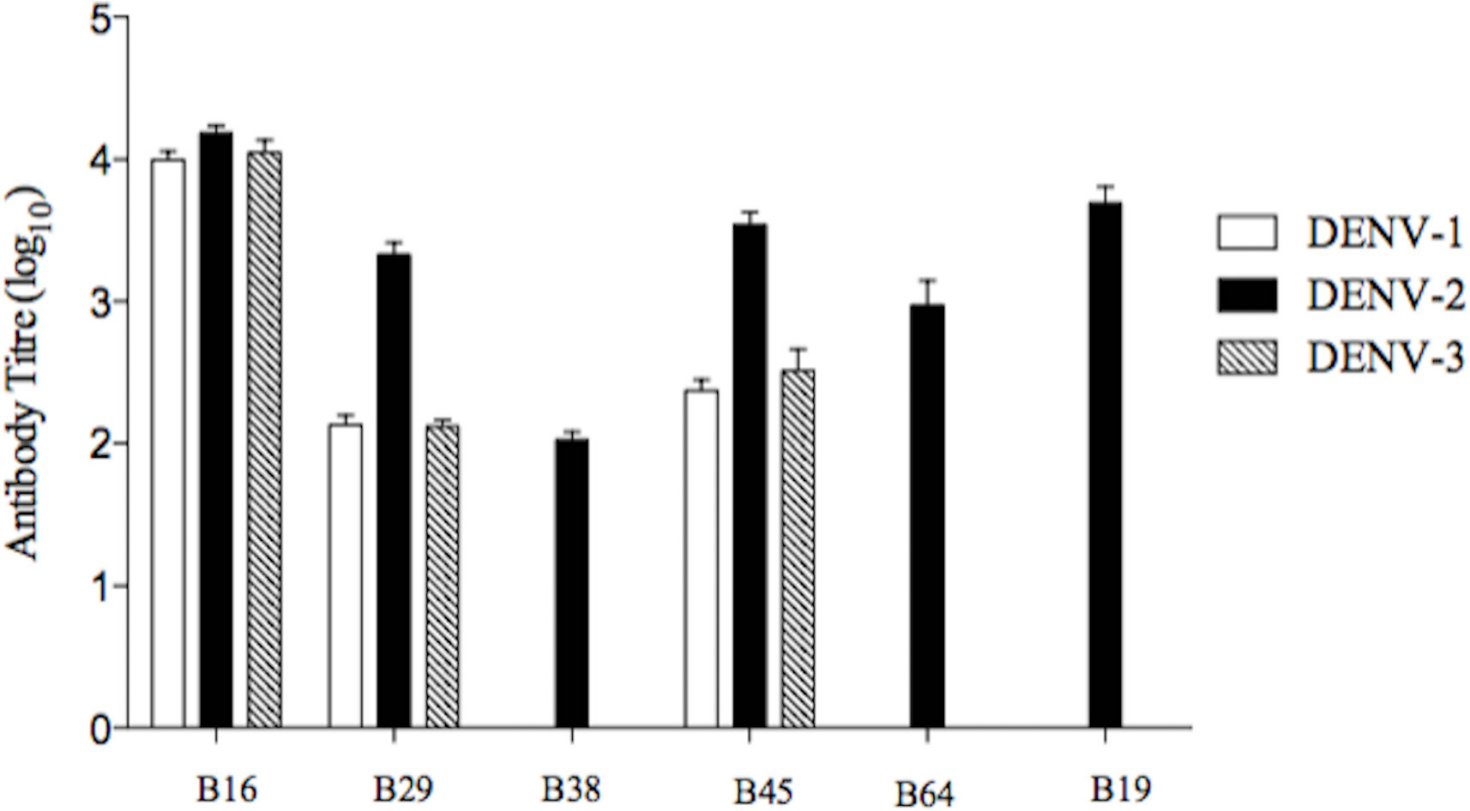
Cross-reactive binding response of anti-peptide antibody against the sE recombinant protein of DENV-1, DENV-2 and DENV-3. Sera raised against each peptide group were pooled (n = 5) and the resulting immune sera were used to test the cross-reactive antibody response in ELISA. The log_10_ antibody titres raised against each vaccine construct (B16, B29, B38, B45, B64 and B19) were evaluated using the recombinant E protein of DENV-1 (clear bar), DENV-2 (dark bar) and DENV-3 (shaded bar).

Sera from all five mice within a group were pooled and the resulting immune sera were used to test the cross-reactive antibody response in ELISA against soluble E (sE) recombinant protein of DENV-1 (395 aa), DENV-2 (395 aa) and DENV-3 (393 aa). The log_10_ antibody titre of six vaccine constructs, B16, B29, B38, B45, B64 and B19, are shown in Fig 6. The construct B2 did not elicit an antibody response to any of the recombinant proteins. All six sera reacted with the DENV-2 recombinant protein. The anti-peptide antibodies were observed to differ widely in their cross-reactivity. The antisera elicited by the vaccine constructs B16, B29 and B45 showed cross-reactivity against all three DENV recombinant proteins (DENV-1, 2 and 3). In particular, the levels of immunoglobulins elicited against the conserved E protein fusion loop represented by construct B16 showed the highest antibody titre and were broadly cross-reactive against DENV-1, DENV-2 and DENV-3 recombinant proteins with log_10_ titres of 4.000, 4.215 and 4.05, respectively. In contrast, three constructs elicited only a homologous antibody response against DENV-2; these were constructs B19 (log_10_ 3.691), B64 (log_10_ 2.975) and B38 (log_10_ 2.025).

### Neutralizing activity of anti-peptide antibodies *in vitro*

**Fig 7:**
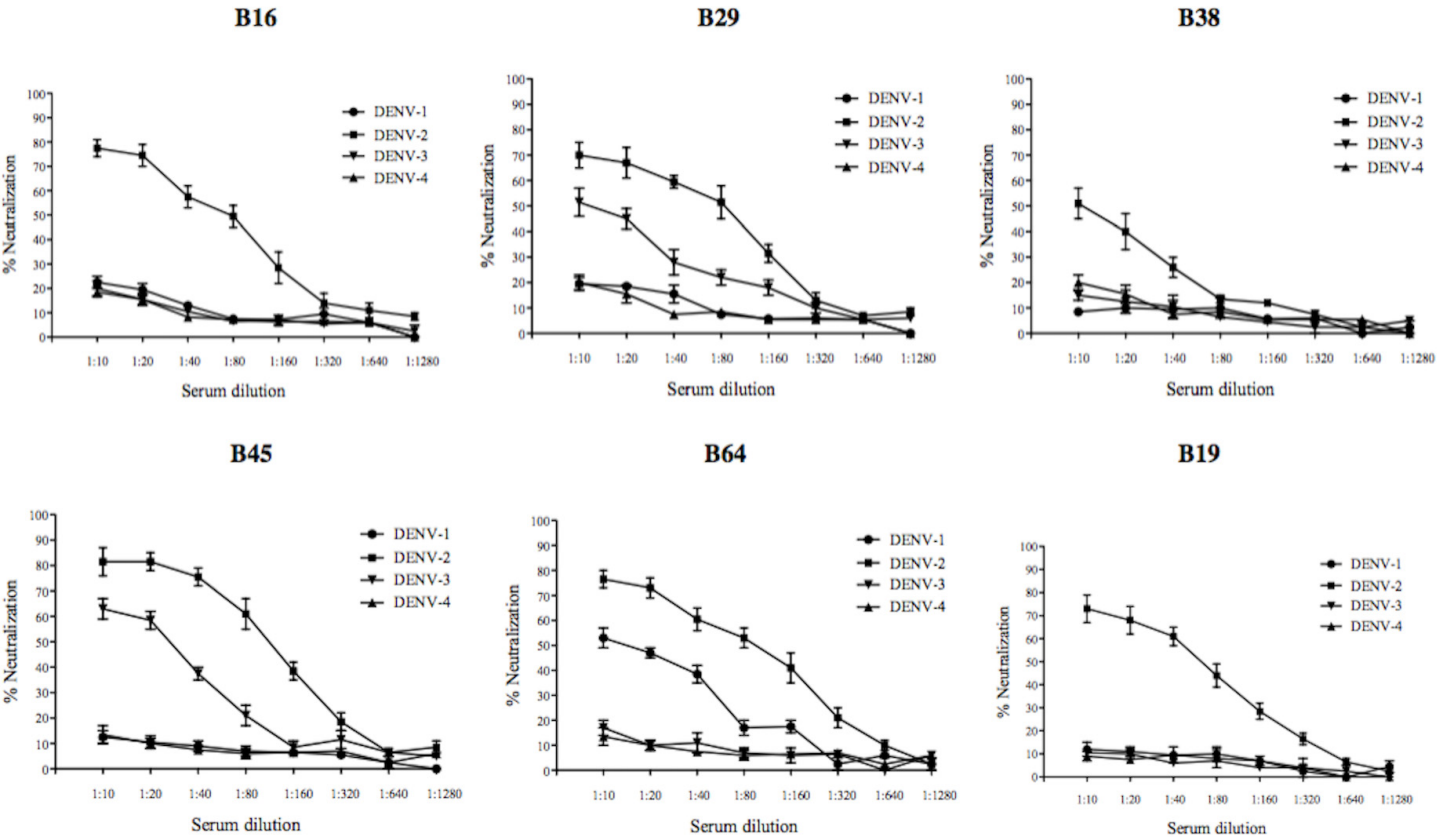
Neutralizing activity of anti-peptide antibodies against four DENV serotypes. Serially diluted pooled immune sera from vaccine constructs B16, B29, B38, B45, B64 and B19 were used to test the virus neutralizing activity against DENV-1, DENV-2, DENV-3 and DENV-4 in an *in vitro* focus reduction neutralization assay (FRNT) employing BHK-21 cells. A dilution resulting in 50% reduction of focus when compared to the pre-immune serum was considered as the end-point titre (FRNT_50_). Each neutralization curve is an average (±S.E.M) of two independent neutralization experiments with a pooled serum group.

Pooled immune sera at various dilutions were used to test the neutralizing activity against DENV-1, DENV-2, DENV-3 and DENV-4 in an *in vitro* focus reduction neutralization assay (FRNT) using BHK-21 cells (Fig 7). The sera dilution which caused a 50% reduction of focus, when compared to the pre-immune serum/saline-adjuvant serum control, was considered to be the end-point titre (FRNT_50_). Homologous neutralizing antibody response against DENV-2 was observed in vaccine constructs B16, B38 and B19 with neutralizing antibody titres (FRNT_50_) of 1:80, 1:10 and 1:40, respectively. The vaccine constructs B29 and B45 showed a heterotypic neutralizing antibody response against both DENV-2 and DENV-3. The 50% neutralizing antibody titres of B29 were 1:80 (DENV-2) and 1:10 (DENV-3), whereas the antibody titres elicited by the B45 vaccine construct against DENV-2 and DENV-3 were 1:80 and 1:20, respectively. The pooled sera from the construct B2 did not elicit a neutralizing antibody response against any of the four DENV serotypes indicating that the antibodies elicited by construct B2 were binding but lacking neutralizing activity. In contrast, the vaccine construct derived from the E ectodomain region represented by B64 elicited a neutralizing antibody response against both DENV-1 and DENV-2 (Fig 6E), with FRNT_50_ titres of 1:10 and 1:80, respectively.

## Discussion

Dengue poses a significant public health threat and currently, no vaccine or antiviral therapy are available. The phase III clinical trials of Sanofi Pasteur tetravalent chimeric yellow-fever dengue vaccine (Dengvaxia^®^) in Asia (Indonesia, Malaysia, Philippines, Thailand, Vietnam) [13] and the Latin America (Brazil, Colombia, Honduras, Mexico, Puerto Rico) [14] showed the overall vaccine efficacies as 56.5% and 64.7%, respectively. However, the serotype-specific vaccine efficacy in Asia was substantially lower at 50% for serotype 1 and 35% for serotype 2 [13]. Similar trend in serotype-specific vaccine efficacy was also reported in the Latin American phase III clinical trial where the efficacies were 50.3% for serotype 1 and 42.3% for serotype 2 [14]. The predominant serotypes circulating in the South East Asian region were reported to be serotype 1 and 2 [41]. Clearly, alternative strategies are needed for the dengue vaccine development pipeline.

Peptide vaccines containing specific epitopes capable of inducing humoral and cellular immune responses are considered to be an alternative strategy [19]. These peptide vaccines can be used for induction of broad-spectrum immunity against multiple strains while eliminating allergenic and/or reactogenic complications [42]. Rocha *et al*. (2014) reported that the tetravalent peptide vaccines derived from the highly conserved region on the dengue E protein elicited antibodies but they showed low neutralizing activity (PRNT_50_ <1:20) and failed to neutralize all 4 serotypes. A possible strategy is that the peptide vaccine constructs can be designed with selected epitopes from the four dengue serotypes [16].

Since DENV serotype 2 and 3 are predominant in Australia, we focused on identifying a B-cell epitope which could elicit cross-reactive neutralizing antibodies against serotypes 2 and 3. Three different epitope mapping strategies were used to identify potential B-cell epitopes on the E protein from dengue virus serotype 2. The soluble E-glycoprotein has three structural domains: EDI (residues 1–52, 132–182 and 280–295), EDII (residues 53–131 and 193–279) and EDIII (residues 303–395) [3]. From the 17 peptides which showed positive binding to IgG by ELISA, those representing amino acid position 106–123 (peptide 16), 197–214 (peptide 29), 225–242 (peptide 33) and 260–277 (peptide 38) are located on EDII and those representing amino acids 295–312 (peptide 43), 309–326 (peptide 45), 323–340 (peptide 47), 330–347 (peptide 48), 365–382 (peptide 53) and 372–389 (peptide 54) are located on EDIII. On the other hand, the solution phase epitope extraction technique revealed 21 peptides that were bound by the immune IgG, of which 9 (peptides 2, 16, 19, 29, 33, 38, 40, 45 and 64) were also identified to be positive in ELISA. These in-solution reactions were useful to maintain the conformational integrity required for some of the peptides to be able to bind to the antibody [25, 43].

A combination of both ELISA and epitope extraction approaches are useful in epitope mapping as these techniques together could enhance the number of epitopes identified. For example, peptides 43, 47, 48, 53, 54, 68, 69 and 70 reacted positively in ELISA only. In contrast, peptides 1, 3, 20, 30, 32, 37, 39, 52, 62, 63, 65 and 66 were identified only in epitope extraction approach. Inclusion of both these mapping strategies could enhance the epitope repertoire when combined together. For instance, peptides 29 and 38 showed mild reactivity against the IgG samples in ELISA whereas; in-solution epitope extraction revealed that these two peptides were able to bind well to several IgG samples. Moreover, peptides 68, 69 and 70 showed positive reactions in ELISA, but did not bind to the antibody in solution. Similar binding reactions in ELISA were seen in DENV-2 trans-membrane domain using murine MAbs [44].

The multi-step *in silico* B-cell epitope prediction approach identified 6 potential antigenic regions on the E protein of DENV-2. These regions encode 16 epitopes predicted to be potential B-cell epitopes. Of 16, two epitopes were not reacted positive in both experimental approaches (aa position 214- LDLPLPWLPGADTQ -227 and 298- SYSMCTGKFKVVKE -311). In addition, a truncated peptide at position 98-DRGWGNGCG -106 was also predicted. Previous computational approaches to identify the epitopes on DENV mostly used a single algorithm to predict the epitopes [17, 22]. Furthermore, most of the predictive epitope data training sets contain data entry ambiguities or error prone alignments with high degree of similarity with other peptide sequences reported within the same data set [26]. Peptides were excluded if they shared sequence homology with human proteins which could induce a deleterious response.

A combination of the three strategies revealed a total of 6 novel peptides (peptides 2, 16, 29, 38, 45 and 64) that could serve as B-cell epitopes. The ELISA and epitope extraction methods are high throughput, cost effective and less time consuming such that the antibody binding potential of synthetic peptides can be rapidly screened. However, when compared to ELISA, the epitope extraction technique requires additional sample preparation steps and technical expertise to perform the peptide identification through mass spectrometry. The computational epitope prediction is quick with epitopes identified using a combination of several predictive algorithms [19]. Hence, these three strategies when combined will be useful for fine epitope mapping of DENV and other related flaviviruses.

Peptide-based vaccines are often poorly immunogenic and require adjuvants and effective delivery systems to improve the immunostimulatory properties. Emulsion based delivery methods are highly adopted in vaccine studies, of which Freund’s complete adjuvant (CFA) and Freund’s incomplete adjuvant (IFA) are most popular [45]. In addition, linear B-cell epitopes are limited in their potential to elicit a strong antibody response as it is challenging to mimic the conformation of native protein [46] and also a lack of helper T-cell response. Using synthetic peptides to induce adequate mature B-cell memory responses often requires cytokines from T-helper (T_H_) cells, and induction of robust helper T-cell response is crucial [47]. Hence when selecting epitopes for B-cell mediated vaccine strategy, we must also focus on induction of helper T-cell responses. T-cell help can be provided through co-synthesizing linear helper T-cell epitopes along with the B-cell epitopes [26].

The 6 peptides identified in this study as potential B-cell epitopes were synthesized along with a T_H_–cell epitope and these vaccine constructs (B2, B16, B29, B38, B45 and B64) were evaluated in inbred BALB/c mice. Peptide 19 was previously shown to recognize anti-dengue sera [39] and we included peptide 19 as a positive control. The vaccine constructs B29 and B45 elicited strong neutralizing response against DENV-2 and a low cross-neutralizing activity against DENV-3. Antibodies elicited by both constructs were able to bind to the E recombinant protein of DENV-1 but was unable to neutralize the DENV-1 serotype. The peptide B29 is located within the EDII domain and nine amino acids were conserved between position 204-KAWLVHRQWF-213 in both DENV-2 and -3. The computational analysis of this region has revealed the highly accessible nature on the surface of the E protein of DENV-2. The EDIII domain has been reported to elicit strong neutralizing response against multiple DENV serotypes [8, 48], and the peptide B45 was located within this domain. In addition to high homotypic neutralizing activity against DENV-2, the construct B-45 elicited heterotypic antibodies that could cross-neutralise DENV-3. Both DENV-2 and -3 serotypes exhibit 10 conserved amino acids between positions 314-ETQHGTIVIRVQY-326. EDIII connects to a stem region and the transmembrane (TM) domain with several epitopes been reported to induce cross-reactive neutralising response [7, 49, 50].

Previous studies have shown that the majority of sequences in the “stem” region were conserved across all DENV serotypes [51] and the epitope B64 representing the “stem” anchor region located at aa 442–459 had 11 conserved amino acids in positions 448-FSGVSWTMKILI-459 between DENV-1 and DENV-2. The computational analysis has revealed that the B64 epitope region is moderately accessible on the surface of the virion particle. The antibodies elicited by B64 were able to neutralize DENV-2 with FRNT_50_ titre 1:80 and DENV-1 at 1:10. Surprisingly, these antibodies reacted against the DENV-2 soluble recombinant protein but did not cross-react with DENV-1. Since the linear amino acid sequence of peptide B64 does not represent any epitope region on the sE protein, the antibody might have bound to a conformational epitope present on DENV-2 but not in other serotypes. This is yet another interesting observation as recent studies have shown that depletion of sE binding human antibodies have minor impact on DENV neutralising ability [52]. In addition, antibodies recognizing multiple domains on the surface of the virion have also been reported [53].

Peptide vaccines are reported to have low immunogenicity when compared to a whole virus or recombinant protein vaccine construct. Low doses of the recombinant truncated E proteins (r80E) of four DENV serotypes manufactured by Hawaii Biotech Inc., HI, USA were shown to induce complete protection in mice and monkeys as measured by PRNT [54]. However, subunit vaccines require bulk culture reactors and purification facilities which questions the cost-effectiveness. Walter Reed Army Institute of Research (WRAIR), Maryland, USA, in collaboration with GlaxoSmithKline Biologicals has evaluated the efficacy of purified inactivated dengue vaccine (PIV) in mice and Rhesus macaques. The candidate tetravalent dengue vaccine formulated with alum or an adjuvant system (AS01, AS03 or AS04) induced robust neutralising antibody titers against all four dengue serotypes [55]. Although inactivated vaccines are free from disadvantages such as reversion to a pathogenic phenotype, they require purification processes for removal of the formaldehyde which may lead to conformation changes of the virion. In addition, due to the need to incorporate four serotypes in the inactivated vaccine, the virus antigen of each serotype may not be equally represented in the vaccine formulation and hence the neutralizing antibodies to each serotype might differ.

Neutralizing antibodies at suboptimal concentrations or pre-existing cross-reactive antibodies in dengue patients are hypothesized to enhance the severity of subsequent DENV infection through the phenomenon known as “Antibody-dependent enhancement (ADE)” of infection in monocytes and macrophages through Fcγ receptor mediated endocytosis [56]. Hence, a successful vaccine must induce sufficiently high levels of heterologous neutralising antibodies to protect against dengue infections and also avoid ADE resulting from future infections. Synthetic peptide vaccines is an attractive alternative strategy as they have a number of potential advantages over conventional vaccines such as (i) the absence of infectious viral material in vaccine formulations, (ii) easy introduction of lipid, carbohydrate and phosphate groups to increase the stability and immunogenicity of vaccine constructs, (iii) economical large-scale production, (iv) ability to store in lyophilized form, eliminating the need for cold-chain, (v) no risk of reversion or recombination (vi) the ability to include multiple vaccine antigenic peptides (epitopes) from several different or the same pathogen [57].

In this study, we showed that mice inoculated with synthetic peptides from the dengue 2 E protein were able to elicit broad neutralising antibodies to either serotypes 1 and 2 (peptide B64) or 2 and 3 (peptides B29 and B45). These peptides were identified using three different epitope mapping approaches and they are conserved within different strains of dengue serotype 2 but also share amino acids with other serotypes. However, the strong neutralizing efficacy was elicited against dengue serotype 2 only and a weak cross-neutralizing response was observed to serotypes 1 and 3. A limitation of the study is that whether the anti-peptide antibodies would pose a greater risk of severe dengue in patients than anti-DENV antibodies arising following a natural infection is unknown. In addition, these peptides may serve as a platform for a monovalent vaccine formulation and similar epitope mapping approaches are required to identify epitopes from serotypes 1, 3 and 4 in order to achieve a balanced high neutralizing titre against all four serotypes in a tetravalent vaccine formulation. Such a vaccine formulation might boost the levels of cross-neutralising antibodies to all four serotypes and combat ADE.

Although the neutralisation titre to the heterologous serotype was lower than the homologous serotype, it has been shown in an earlier study that human subjects who received a primary dose of monovalent live attenuated dengue vaccine elicited a strong serotype specific response and low levels of heterotypic cross-neutralizing antibody response. However, upon secondary immunization with a heterotypic monovalent vaccine, it was able to elicit high levels of neutralising antibody response to all four serotypes. In particular, individuals developed multitypic neutralizing antibodies not only against the exposed serotypes but also against serotypes to which they have not yet been exposed. These heterotypic antibodies could protect against multiple DENV serotypes and avoid ADE [58]. Clearly, further investigation is required to evaluate the ADE effect of the cross-neutralizing epitopes identified in our approach and the effect of mutations within these epitope sequences.

A successful vaccine must elicit both humoral and cellular immune responses against all four serotypes, and protect against the disease even upon multiple infections. The epitopes identified in our approach in conjunction with other well-documented epitopes of DENV have implications for future development of epitope-based synthetic dengue vaccines. It is crucial to develop a tetravalent dengue vaccine that elicits specific neutralizing antibodies against all four DENV serotypes to overcome the phenomenon of antibody- dependent enhancement associated with DSS and DHF [1, 10]. Since vaccination with a tetravalent vaccine appears to be a promising strategy for dengue disease prevention, the combined approaches employed in the current investigation could be used to evaluate the potential B-cell epitopes against all four DENV serotypes. These serotype specific epitopes may be combined for the development of a future tetravalent multi-epitope vaccine comprising selected epitope sequences from all four dengue serotypes.

## Supporting Information

S1 Fig Binding of anti-peptide antibodies to the conjugate peptide containing both T-helper and B-cell epitope. (DOCX)

S1 Table Neutralizing antibody response of DENV positive human IgG against 4 DENV prototype strains. (DOCX)

S2 Table ELISA showing peptides against anti-dengue human IgG. (DOCX)

S3 Table Peptides reacting against anti-dengue human IgG determined by epitope extraction. (DOCX)
