# Supplementary material for: Synthetic B-Cell Epitopes Eliciting Cross-Neutralizing Antibodies: Strategies for Future Dengue Vaccine
Source: PLoS One. 2016 May 25;11(5):e0155900. doi: 10.1371/journal.pone.0155900 (PMC4880327; doi:10.1371/journal.pone.0155900)
Supplement: S1 Fig — (DOCX) [file pone.0155900.s001.docx]

**S1 Fig. Binding of anti-peptide antibodies to the conjugate peptide containing both T-helper and B-cell epitope.** For primary (open circles) inoculation, groups of BALB/c mice (n=5) were administered subcutaneously with 50 μg of the peptide immunogen in complete Freund’s adjuvant (CFA) on day 0 (1^o^). Negative control animal groups received CFA and saline. The secondary (closed circles) booster dose was administered subcutaneously on day 28 (2^o^) with 50 μg of the peptide immunogen in incomplete Freund’s adjuvant (IFA). Mice were bled on days 0, 10 and 38, and sera obtained. ELISA was performed using the corresponding conjugate peptide (both T-helper and B-cell peptide) as antigen coated overnight on 96 well plates. Antibody titres are expressed as the reciprocal of the logarithm of that dilution of serum that gave an optical density four times above that obtained in wells with pre-immune control sera. Individual animal titres are presented with the mean value represented by the horizontal bar.
