# Supplementary material for: Synthetic B-Cell Epitopes Eliciting Cross-Neutralizing Antibodies: Strategies for Future Dengue Vaccine
Source: PLoS One. 2016 May 25;11(5):e0155900. doi: 10.1371/journal.pone.0155900 (PMC4880327; doi:10.1371/journal.pone.0155900)
Supplement: S1 Table — (DOCX) [file pone.0155900.s002.docx]

**S1 Table. Neutralizing antibody response of DENV positive human IgG against 4 DENV prototype strains.**

| Sera sample ID | Age  (years) | Infection/ sample collection Interval  (years) | Place of infection | Infected DENV  serotype | Log_10_ neutralization index* against virus serotype | | | | Primary or Secondary  Infection |
| --- | --- | --- | --- | --- | --- | --- | --- | --- | --- |
|  |  |  |  |  | DENV-1 | DENV-2 | DENV-3 | DENV-4 |  |
| 2 | 53 | 9 | Cairns, Australia | 2 | 0.125 | 2.300 | 0.902 | 0.204 | Primary |
| 15 | 51 | 1 | Borneo | 2 | 0.124 | 3.069 | 0.928 | 0.894 | Primary |
| 18 | 55 | 7 | Cairns, Australia | 2 | 0.146 | 1.941 | 0.844 | 0.191 | Primary |
| 19 | 52 | 17 | Townsville, Australia | 2 | 0.360 | 2.013 | 1.016 | 0.128 | Primary |
| 24 | 67 | 6 | Portsmith, Australia | 2 | 0.204 | 3.358 | 0.892 | 0.463 | Primary |
| 27 | 71 | 33 | Papua New Guinea | 2 | 0.096 | 1.795 | 0.493 | 0.192 | Primary |
| 29 | 47 | 17 | Bungalow, Australia | 2 | 0.449 | 2.129 | 0.412 | 0.190 | Primary |
| 31 | 62 | 25 | Papua New Guinea | 2 | 0.283 | 2.356 | 0.397 | 0.096 | Primary |
| 33 | 35 | 1 | Moorooboo, Australia | 2 | 0.919 | 1.939 | 0.928 | 0.384 | Primary |
| 3 | 57 | 5 | Cairns, Australia | 2 | 0.968 | 3.107 | 2.171 | 0.269 | Secondary |
| 17 | 84 | 7 | Cairns, Australia | 2 | 0.286 | 1.110 | 0.190 | 0.235 | Secondary |
| 25 | 50 | 10 | Thailand | 2 | 0.146 | 1.242 | 0.543 | 1.146 | Secondary |
| 39 | 65 | 5 | Machans Beach, Australia | 2 | 0.190 | 1.449 | 0.655 | 1.645 | Secondary |
| 1 | 50 | 2 | Cairns, Australia | 3 | 0.158 | 0.928 | 2.970 | 0.943 | Primary |
| 4 | 84 | 1 | Cairns, Australia | 3 | 0.230 | 0.874 | 1.691 | 0.096 | Primary |
| 5 | 62 | 37 | Singapore | 3 | 0.420 | 0.818 | 1.161 | 0.161 | Primary |
| 7 | 64 | 1 | Cairns, Australia | 3 | 0.230 | 0.952 | 1.868 | 0.169 | Primary |
| 8 | 45 | 2 | Cairns, Australia | 3 | 0.770 | 0.972 | 1.990 | 0.435 | Primary |
| 9 | 57 | 1 | Cairns, Australia | 3 | 0.322 | 0.988 | 2.045 | 0.928 | Primary |
| 12 | 42 | 1 | Cairns, Australia | 3 | 0.481 | 1.083 | 2.951 | 0.451 | Primary |
| 13 | 85 | 1 | Cairns, Australia | 3 | 0.212 | 0.888 | 1.344 | 0.491 | Primary |
| 22 | 68 | 2 | Port Douglas, Australia | 3 | 0.190 | 0.411 | 1.118 | 0.587 | Primary |
| 23 | 66 | 2 | Port Douglas, Australia | 3 | 0.176 | 0.522 | 1.134 | 0.363 | Primary |
| 28 | 69 | 1 | Edgehill, Australia | 3 | 0.130 | 0.839 | 3.390 | 0.389 | Primary |
| 35 | 52 | 3 | Stratford, Australia | 3 | 0.146 | 0.543 | 3.601 | 0.602 | Primary |
| 36 | 57 | 2 | Whitfield, Australia | 3 | 0.272 | 0.920 | 1.573 | 0.176 | Primary |
| 40 | 35 | 2 | Cairns, Australia | 3 | 0.081 | 0.818 | 1.257 | 0.859 | Primary |
| 6 | 79 | 2 | Cairns, Australia | 3 | 0.272 | 1.176 | 1.272 | 0.176 | Secondary |
| 14 | 71 | 6 | Cairns, Australia | 3 | 0.139 | 1.257 | 1.859 | 1.285 | Secondary |
| 16 | 46 | 1 | Cairns, Australia | 3 | 1.100 | 1.859 | 1.818 | 1.161 | Secondary |
| 20 | 64 | 1 | Cairns, Australia | 3 | 0.750 | 1.262 | 1.496 | 1.111 | Secondary |
| 30 | 78 | 1 | Earlville, Australia | 3 | 0.190 | 0.491 | 2.286 | 0.587 | Secondary |
| 32 | 65 | 1 | Cairns, Australia | 3 | 1.216 | 1.844 | 2.367 | 0.146 | Secondary |
| 34 | 51 | 1 | Kewarra Beach, Australia | 3 | 0.190 | 0.805 | 1.817 | 0.493 | Secondary |
| 37 | 56 | 24 | Tahiti | 1 | 2.000 | 0.348 | 0.285 | 0.558 | Primary |

* A log neutralization index of 1 was considered as a cut-off value for positive neutralization
