# Supplementary material for: Synthetic B-Cell Epitopes Eliciting Cross-Neutralizing Antibodies: Strategies for Future Dengue Vaccine
Source: PLoS One. 2016 May 25;11(5):e0155900. doi: 10.1371/journal.pone.0155900 (PMC4880327; doi:10.1371/journal.pone.0155900)
Supplement: S2 Table — (DOCX) [file pone.0155900.s003.docx]

**S2 Table. ELISA showing peptides against anti-dengue human IgG**

| **Peptide number** | **Sera number** | | | | | | | | | | | | | | | | | |
| --- | --- | --- | --- | --- | --- | --- | --- | --- | --- | --- | --- | --- | --- | --- | --- | --- | --- | --- |
|  | **2** | **15** | **18** | **19** | **24** | **27** | **29** | **31** | **33** | **3** | **17** | **25** | **39** | **1** | **4** | **5** | **7** | **8** |
| **2** | 0.522 | 0.854 | 0.070 | 0.253 | 1.131 | 0.074 | 0.578 | 0.572 | 0.551 | 0.798 | 0.457 | 0.450 | 0.473 | 0.381 | 0.216 | 0.375 | 0.367 | 0.177 |
| **16** | 0.608 | 1.390 | 0.413 | 1.132 | 1.203 | 0.548 | 1.023 | 1.048 | 0.944 | 1.547 | 0.803 | 0.779 | 0.693 | 0.834 | 1.055 | 0.942 | 1.339 | 0.611 |
| **19** | 0.916 | 0.618 | 0.235 | 0.406 | 0.521 | 0.752 | 0.318 | 0.352 | 0.288 | 0.899 | 0.192 | 0.226 | 0.232 | 0.516 | 0.406 | 0.089 | 0.668 | 0.325 |
| **29** | 0.086 | 0.199 | 0.113 | 0.299 | 0.046 | 0.074 | 0.071 | 0.108 | 0.069 | 0.205 | 0.088 | 0.089 | 0.057 | 0.036 | 0.066 | 0.071 | 0.076 | 0.093 |
| **33** | 0.042 | 0.083 | 0.119 | 0.250 | 0.840 | 0.100 | 0.094 | 0.089 | 0.112 | 0.189 | 0.095 | 0.072 | 0.052 | 0.094 | 0.082 | 0.057 | 0.069 | 0.121 |
| **38** | 0.340 | 0.251 | 0.101 | 0.228 | 0.291 | 0.064 | 0.084 | 0.091 | 0.048 | 0.191 | 0.072 | 0.082 | 0.090 | 0.081 | 0.058 | 0.090 | 0.076 | 0.116 |
| **40** | 0.484 | 0.816 | 0.305 | 0.854 | 0.629 | 0.048 | 0.497 | 0.418 | 0.548 | 0.720 | 0.323 | 0.372 | 0.512 | 0.635 | 0.858 | 0.571 | 0.920 | 0.369 |
| **43** | 0.065 | 0.343 | 0.110 | 0.434 | 0.118 | 0.056 | 0.055 | 0.082 | 0.211 | 0.344 | 0.071 | 0.123 | 0.187 | 0.087 | 0.055 | 0.062 | 0.086 | 0.203 |
| **45** | 1.279 | 0.992 | 0.693 | 0.634 | 0.048 | 0.493 | 0.456 | 0.412 | 0.555 | 0.840 | 0.163 | 0.349 | 0.299 | 1.181 | 0.913 | 0.325 | 1.154 | 0.740 |
| **47** | 0.863 | 0.200 | 0.461 | 0.103 | 0.051 | 0.070 | 0.085 | 0.080 | 0.117 | 0.135 | 0.270 | 0.069 | 0.147 | 0.057 | 0.430 | 0.055 | 0.858 | 0.420 |
| **48** | 0.605 | 0.187 | 0.219 | 0.043 | 0.051 | 0.046 | 0.072 | 0.056 | 0.431 | 0.145 | 0.112 | 0.155 | 0.047 | 0.107 | 0.246 | 0.048 | 0.263 | 0.169 |
| **53** | 0.539 | 0.048 | 0.361 | 0.361 | 0.540 | 0.065 | 0.215 | 0.046 | 0.070 | 0.695 | 0.068 | 0.178 | 0.076 | 0.412 | 0.065 | 0.069 | 0.423 | 0.270 |
| **54** | 0.752 | 0.550 | 0.256 | 0.410 | 0.568 | 0.259 | 0.284 | 0.242 | 0.271 | 0.836 | 0.227 | 0.245 | 0.061 | 0.436 | 0.378 | 0.038 | 0.397 | 0.221 |
| **64** | 0.562 | 0.598 | 0.460 | 0.498 | 0.702 | 0.392 | 0.286 | 0.322 | 0.394 | 0.838 | 0.285 | 0.206 | 0.267 | 0.628 | 0.554 | 0.262 | 0.552 | 0.340 |
| **68** | 0.609 | 0.270 | 0.483 | 0.208 | 0.448 | 0.046 | 0.096 | 0.110 | 0.107 | 0.336 | 0.093 | 0.112 | 0.067 | 0.054 | 0.517 | 0.090 | 0.497 | 0.499 |
| **69** | 0.551 | 0.615 | 0.416 | 0.421 | 0.498 | 0.056 | 0.052 | 0.115 | 0.155 | 0.742 | 0.111 | 0.120 | 0.095 | 0.572 | 0.482 | 0.086 | 0.565 | 0.434 |
| **70** | 0.757 | 0.904 | 0.641 | 0.616 | 0.501 | 0.045 | 0.117 | 0.118 | 0.045 | 1.068 | 0.098 | 0.118 | 0.088 | 0.917 | 0.706 | 0.098 | 0.843 | 0.597 |

**S2 Table continuation.**

| **Peptide number** | **Sera number** | | | | | | | | | | | | | | | |  |
| --- | --- | --- | --- | --- | --- | --- | --- | --- | --- | --- | --- | --- | --- | --- | --- | --- | --- |
|  | **9** | **12** | **13** | **22** | **23** | **28** | **35** | **36** | **40** | **6** | **14** | **16** | **20** | **30** | **32** | **34** | **37** |
| **2** | 0.417 | 0.592 | 0.075 | 0.268 | 0.206 | 0.596 | 0.998 | 0.404 | 0.378 | 0.060 | 0.287 | 0.647 | 0.190 | 0.698 | 0.478 | 0.562 | 0.611 |
| **16** | 0.852 | 1.254 | 0.382 | 0.800 | 0.725 | 0.682 | 0.838 | 0.611 | 0.389 | 0.647 | 0.605 | 0.690 | 0.601 | 0.908 | 0.858 | 0.490 | 1.069 |
| **19** | 0.367 | 0.512 | 0.064 | 0.190 | 0.464 | 0.495 | 0.305 | 0.292 | 0.202 | 0.341 | 0.322 | 0.425 | 0.264 | 0.413 | 0.389 | 0.195 | 0.327 |
| **29** | 0.049 | 0.123 | 0.046 | 0.082 | 0.058 | 0.093 | 0.110 | 0.061 | 0.107 | 0.170 | 0.115 | 0.216 | 0.156 | 0.077 | 0.060 | 0.044 | 0.075 |
| **33** | 0.076 | 0.042 | 0.061 | 0.093 | 0.264 | 0.088 | 0.075 | 0.046 | 0.306 | 0.101 | 0.150 | 0.074 | 0.178 | 0.098 | 0.120 | 0.190 | 0.117 |
| **38** | 0.072 | 0.056 | 0.068 | 0.062 | 0.098 | 0.090 | 0.124 | 0.223 | 0.109 | 0.117 | 0.087 | 0.145 | 0.134 | 0.089 | 0.111 | 0.069 | 0.072 |
| **40** | 0.403 | 0.589 | 0.062 | 0.581 | 0.347 | 0.372 | 0.641 | 0.603 | 0.987 | 0.578 | 0.384 | 0.417 | 0.573 | 0.359 | 0.417 | 0.385 | 0.679 |
| **43** | 0.097 | 0.354 | 0.109 | 0.118 | 0.242 | 0.086 | 0.105 | 0.253 | 0.224 | 0.443 | 0.093 | 0.056 | 0.102 | 0.161 | 0.178 | 0.223 | 0.092 |
| **45** | 0.475 | 0.755 | 0.239 | 0.050 | 0.695 | 0.544 | 0.501 | 0.491 | 0.372 | 0.934 | 1.107 | 0.596 | 0.745 | 0.511 | 0.476 | 0.342 | 0.508 |
| **47** | 0.070 | 0.058 | 0.062 | 0.235 | 0.113 | 0.125 | 0.077 | 0.062 | 0.062 | 0.468 | 0.063 | 0.210 | 0.129 | 0.183 | 0.154 | 0.062 | 0.089 |
| **48** | 0.083 | 0.071 | 0.067 | 0.068 | 0.119 | 0.111 | 0.068 | 0.037 | 0.543 | 0.265 | 0.052 | 0.154 | 0.076 | 0.068 | 0.169 | 0.050 | 0.080 |
| **53** | 0.088 | 0.337 | 0.093 | 0.085 | 0.392 | 0.387 | 0.287 | 0.282 | 0.085 | 0.443 | 0.757 | 0.305 | 0.302 | 0.341 | 0.330 | 0.094 | 0.302 |
| **54** | 0.262 | 0.428 | 0.055 | 0.303 | 0.456 | 0.406 | 0.260 | 0.046 | 0.053 | 0.347 | 0.343 | 0.357 | 0.260 | 0.316 | 0.292 | 0.050 | 0.278 |
| **64** | 0.352 | 0.590 | 0.047 | 0.360 | 0.310 | 0.423 | 0.336 | 0.306 | 0.297 | 0.506 | 0.468 | 0.381 | 0.382 | 0.318 | 0.419 | 0.235 | 0.458 |
| **68** | 0.120 | 0.083 | 0.067 | 0.070 | 0.078 | 0.060 | 0.091 | 0.103 | 0.059 | 0.524 | 0.074 | 0.171 | 0.090 | 0.111 | 0.222 | 0.081 | 0.270 |
| **69** | 0.292 | 0.490 | 0.039 | 0.083 | 0.248 | 0.083 | 0.113 | 0.274 | 0.080 | 0.477 | 0.534 | 0.352 | 0.268 | 0.144 | 0.236 | 0.184 | 0.265 |
| **70** | 0.442 | 0.669 | 0.096 | 0.076 | 0.262 | 0.094 | 0.109 | 0.245 | 0.069 | 0.701 | 0.853 | 0.482 | 0.145 | 0.131 | 0.205 | 0.188 | 0.283 |

Samples of IgG at a concentration of 20 µg/ml were used in direct binding ELISA to test the cross reactivity against peptides and the data are presented as the mean optical density of triplicates. IgG from 4 non-infected individuals were used as a negative control and the cut-off value to select the positive peptides was OD-0.128. The positive peptides were selected based on the antibody reaction above the cut-off value.
