# Supplementary material for: Synthetic B-Cell Epitopes Eliciting Cross-Neutralizing Antibodies: Strategies for Future Dengue Vaccine
Source: PLoS One. 2016 May 25;11(5):e0155900. doi: 10.1371/journal.pone.0155900 (PMC4880327; doi:10.1371/journal.pone.0155900)
Supplement: S3 Table — (DOCX) [file pone.0155900.s004.docx]

**S3 Table. Peptides reacting against anti-dengue human IgG determined by epitope extraction**

| **Peptides** | **Sera number** | | | | | | | | | | | | | | | | | | | | | | | | | | | | | | | | | | | |
| --- | --- | --- | --- | --- | --- | --- | --- | --- | --- | --- | --- | --- | --- | --- | --- | --- | --- | --- | --- | --- | --- | --- | --- | --- | --- | --- | --- | --- | --- | --- | --- | --- | --- | --- | --- | --- |
|  | **2** | **15** | **18** | **19** | **24** | **27** | **29** | **31** | **33** | **3** | **17** | **25** | **39** | **1** | **4** | **5** | **7** | **8** | **9** | **12** | **13** | **22** | **23** | **28** | **35** | **36** | **40** | **6** | **14** | **16** | **20** | **30** | **32** | **34** | **37** |  |
| **1** | + |  |  | + |  |  |  | + | + | + |  | + |  | + |  |  | + | + |  |  |  |  | + |  |  | + |  | + |  | + |  |  | + |  | + |  |
| **2** | + | + |  | + | + |  | + | + | + | + | + | + | + | + | + | + | + | + | + | + |  | + | + | + | + | + | + |  | + | + | + | + | + | + | + |  |
| **3** | + | + |  | + | + | + |  | + | + | + | + | + | + | + | + | + | + | + | + | + |  | + |  |  | + | + | + | + | + | + | + | + | + | + | + |  |
| **16** | + | + | + | + | + | + | + | + | + | + | + | + | + | + | + | + | + | + | + | + | + | + | + | + | + |  |  |  | + | + | + | + | + | + | + |  |
| **19** | + | + | + | + | + | + | + | + | + | + | + | + | + | + | + |  | + | + | + | + |  | + | + | + | + | + | + | + | + | + | + | + | + | + | + |  |
| **20** | + | + |  | + |  | + |  | + | + | + | + | + |  | + | + | + | + |  |  | + |  | + |  |  | + | + | + | + |  | + |  | + | + | + | + |  |
| **29** | + | + | + | + | + | + | + | + | + | + | + | + | + | + | + | + | + | + | + | + | + | + | + | + | + | + | + | + | + | + | + | + | + | + | + |  |
| **30** |  | + |  | + | + | + |  | + | + | + | + | + | + | + | + | + | + | + | + | + | + | + | + | + | + | + | + | + |  | + | + | + |  | + | + |  |
| **32** | + | + |  | + |  | + |  | + | + | + |  | + |  | + | + |  | + |  |  | + |  | + |  |  |  |  | + |  |  | + |  | + | + | + | + |  |
| **33** |  |  |  | + |  | + |  |  |  |  |  | + |  |  | + |  | + |  |  | + |  | + |  |  |  | + | + | + |  | + |  |  |  | + | + |  |
| **37** |  | + |  |  | + |  |  | + | + | + | + | + | + | + | + | + | + |  | + |  |  | + |  |  | + | + | + | + |  | + | + | + |  | + | + |  |
| **38** | + | + | + | + | + | + | + | + | + | + | + | + | + | + | + | + | + | + | + | + |  | + | + |  | + | + | + | + | + | + | + |  |  | + | + |  |
| **39** |  |  | + | + | + | + | + | + | + | + | + | + | + | + | + | + | + | + | + | + |  | + | + |  | + | + | + | + | + | + | + |  |  | + | + |  |
| **40** |  |  | + | + | + | + |  | + | + | + | + | + | + | + | + | + | + | + | + |  |  | + | + |  | + | + | + | + | + | + | + |  |  | + | + |  |
| **45** | + | + | + | + | + | + | + | + | + | + | + | + | + | + | + | + | + | + | + | + | + | + | + | + | + | + | + | + | + | + | + | + | + | + | + |  |
| **52** |  | + |  | + |  | + |  | + | + | + | + | + |  | + | + | + | + |  |  | + |  | + |  |  | + | + | + | + |  | + |  | + |  | + | + |  |
| **62** | + | + |  | + |  | + |  | + | + | + |  | + |  | + | + |  | + |  |  | + |  | + |  |  |  |  | + |  |  | + |  | + | + |  | + |  |
| **63** | + | + | + | + |  | + |  | + | + | + |  | + |  | + | + |  | + | + |  | + |  | + | + | + | + | + | + | + | + | + | + | + | + | + | + |  |
| **64** | + | + | + | + | + | + | + | + | + | + | + | + |  | + |  | + | + | + |  | + |  | + | + |  | + |  | + |  | + | + |  | + | + |  | + |  |
| **65** |  |  | + | + | + | + | + | + | + |  |  | + | + |  |  |  |  |  |  |  |  | + | + |  |  | + | + |  |  | + | + | + | + | + | + |  |
| **66** | + |  | + | + |  | + |  | + | + | + |  | + |  | + | + |  | + | + | + | + |  |  |  |  |  | + | + | + | + | + | + |  |  | + |  |  |

Pools of overlapping peptides (18-mers, n=5) spanning the entire E protein of DENV-2 were assembled such that no pool contained peptides of similar mass. These peptide pools were allowed to react with IgG purified from sera of DENV-2 infected subjects and the antibody-bound peptides were extracted in acidic conditions. Following extraction, the bound peptides were identified through MALDI-ToF mass spectrometry. + indicates positive binding of peptides to IgG.
